# Energy demand and the context‐dependent effects of genetic interactions underlying metabolism

**DOI:** 10.1002/evl3.47

**Published:** 2018-04-03

**Authors:** Luke A. Hoekstra, Cole R. Julick, Katelyn M. Mika, Kristi L. Montooth

**Affiliations:** ^1^ Department of Evolution, Ecology and Organismal Biology Iowa State University Ames Iowa 50011; ^2^ School of Biological Sciences University of Nebraska‐Lincoln Lincoln Nebraska 68588; ^3^ Department of Human Genetics University of Chicago Chicago Illinois 60637

**Keywords:** *Drosophila melanogaster*, energetics, epistasis, gene‐environment interaction, life‐history tradeoffs, metabolic rate, mtDNA, phenotypic plasticity

## Abstract

Genetic effects are often context dependent, with the same genotype differentially affecting phenotypes across environments, life stages, and sexes. We used an environmental manipulation designed to increase energy demand during development to investigate energy demand as a general physiological explanation for context‐dependent effects of mutations, particularly for those mutations that affect metabolism. We found that increasing the photoperiod during which *Drosophila* larvae are active during development phenocopies a temperature‐dependent developmental delay in a mitochondrial‐nuclear genotype with disrupted metabolism. This result indicates that the context‐dependent fitness effects of this genotype are not specific to the effects of temperature and may generally result from variation in energy demand. The effects of this genotype also differ across life stages and between the sexes. The mitochondrial‐nuclear genetic interaction disrupts metabolic rate in growing larvae, but not in adults, and compromises female, but not male, reproductive fitness. These patterns are consistent with a model where context‐dependent genotype‐phenotype relationships may generally arise from differences in energy demand experienced by individuals across environments, life stages, and sexes.

**Impact Summary**

Genetic effects on traits are often context dependent, such that a genotype that improves fitness under one context may have no effect or even a deleterious effect in another context. The external environment is a common context that affects the degree to which a genotype determines a phenotype, but the internal environment of an organism (e.g., its genetic background, sex, or life stage) also provides an important context that may modify the phenotypic expression of a genotype. Here, we combine new data on the phenotypic effects of a well‐characterized genetic interaction between the mitochondrial and nuclear genomes of the fruit fly *Drosophila* with prior observations to support a model of energy demand as a general explanation for context‐dependent genetic effects, particularly for mutations that affect metabolism. We show that the magnitude of fitness effects of this genetic interaction correlates positively with the degree of energy demand among developmental treatments that accelerate growth rate, across developmental stages that differ in the cost of growth, and between sexes with potentially different costs of reproduction. These internal and external contexts create variable demands on energy metabolism that will impact the efficacy of natural selection acting on metabolic mutations in populations.

Environment, development, and physiological state can all modify the phenotypic expression of genetic variation (e.g., Hartman et al. 2001; Raj et al. 2010). Because natural selection acts upon the subset of expressed genetic variation that affects fitness, the fate of new mutations may depend on the landscape of genetic backgrounds and environments experienced by a population (e.g., Remold and Lenski 2004; Chandler et al. 2013; Lachance et al. 2013; Wang et al. 2013; Kammenga, 2017). Routine variation in the internal—genetic, developmental, physiological—or external environment can challenge the capacity of individuals to maintain homeostasis, and this can magnify deleterious mutational effects (e.g., Kondrashov and Houle 1994; Hoekstra et al. 2013). Conversely, favorable environments can mask potential genotype‐phenotype relationships (Harshman and Zera 2007; Agrawal et al. 2010; Hoekstra et al. 2013). If the relationship between genotype and fitness is generally conditional on internal or external environmental factors (i.e., is context dependent), then elucidating general principles underlying genotype‐phenotype–environment interactions is critical for understanding evolutionary processes such as the maintenance of genetic variation for life‐history traits (Roff and Fairbairn 2007; Van Dyken and Wade 2010; Mackay 2014).

If context‐dependent genetic effects mediate phenotypic tradeoffs—often manifest as negative phenotypic trait correlations—then dissecting the underlying physiology can provide mechanistic explanations of phenotypic correlations that better enable predictions regarding the performance of particular genotypes in particular environments (Harshman and Zera 2007; Flatt and Heyland 2011). Many phenotypic tradeoffs likely result from the differential allocation of finite resources to growth, survival, and reproduction (Van Noordwijk and Dejong 1986; Roff 2002). Traits such as growth rate and gamete production demand sufficient energy production supplied by metabolic processes, but the rate of metabolism itself is subject to homeostatic regulation that can influence energy allocation and obscure trait correlations (Clarke and Fraser 2004; Harshmann and Zera 2007; Leopold and Perrimon 2007). While many good examples of the importance of environmental context for tradeoffs exist (reviewed in Asplen et al. 2012), understanding the genetic architecture underlying tradeoffs and the physiological mechanisms mediating them lags behind (Roff and Fairbairn 2007).

To begin to fill this gap in our understanding, we combined well‐characterized mitochondrial‐nuclear (hereinafter mito‐nuclear) genotypes that affect metabolism, physiology, and fitness in Drosophilid flies (Hoekstra et al. 2013; Meiklejohn et al. 2013; Holmbeck et al. 2015; Zhang et al. 2017) with an environmental perturbation designed to manipulate energy demand. Phenotypic effects of variation in the mitochondrial genome (mtDNA) often depend upon variation in the nuclear genome due to the functional interactions between gene products from these two genomes (reviewed in Burton and Barreto 2012). In ectotherms, the phenotypic effects of these mito‐nuclear genetic interactions frequently depend upon temperature (Dowling et al. 2007a; Arnqvist et al. 2010; Hoekstra et al. 2013; Paliwal et al. 2014). For example, cool development temperatures masked the deleterious effects of a mito‐nuclear incompatibility in *Drosophila*, while warmer temperatures generated inefficiencies in larval metabolism that magnified the deleterious effects of the incompatibility on development rate (Hoekstra et al. 2013). We proposed that this was due to the accelerating effect of temperature on development rate and increasing demand on energetic processes during rapid larval growth. However, to distinguish direct, thermodynamic effects of temperature on interacting mito‐nuclear gene products from more generic effects of variation in energetic demand, we sought to manipulate developmental rate independent of temperature. In many insects, including *Drosophila*, endogeneous circadian clocks influence the timing of developmental hallmarks (e.g., pupation, eclosion; Kyriacou et al. 1990). Circadian rhythms result in “gated” periods of development, which can be entrained by diurnal variation in photoperiod to further increase the synchronicity of discrete developmental events (e.g., pupation, eclosion; Ashmore and Sehgal 2003, Nijhout et al. 2010; Yadav et al. 2014). Flies can develop fastest in constant light, absent of photoperiodic entrainment (Paranjpe et al. 2005). We hypothesized that the presence of photoperiodic entrainment in Hoekstra et al. (2013) may have synchronized development, constraining the effects of variation in mito‐nuclear compatibility on development rate. Here, we show that manipulating the developmental photoperiod to accelerate growth, which putatively generates increased demand on the energy metabolism required to supply rapid growth, generates similar context‐dependent effects of this genotype in delaying development that are independent of temperature. This suggests that the balance of energy supply via metabolism and energy demand—the metabolic costs associated with maintenance, growth, and organismal performance—may be a general physiological explanation for why some environments expose, while others mask, genetic effects.

Energy demand may also provide a general explanation for why genetic effects vary across life stages and between sexes. The substantial metabolic cost of growth (Parry 1983; Glazier 2005) may cause the energy budget of developing organisms to be more constrained than that of adults. This may be particularly true for holometabolous insects that experience exponential growth during development before reaching a relatively static adult size (Church and Robertson, 1966). Thus, a genotype that has inefficient energy metabolism may be able to meet energy demands for maintenance as an adult (with an adult metabolism), but not as a larvae (with a larval metabolism). After the cessation of growth, adults of different sexes may partition energy in different ways due to the differential costs of reproduction (Bateman 1948; Hayward and Gillooly 2011), potentially generating sex‐specific effects of mutations. Here, we present patterns of context‐dependent effects of a mito‐nuclear incompatibility that are consistent with a model where internal and external environments that cause energy demand to exceed supply may generally expose mutational effects on phenotypes. This has important consequences for the efficacy of natural selection acting on mutations in populations, and particularly for those mutations that impact metabolism.

## Methods

### DROSOPHILA *GENOTYPES*

We used mito‐nuclear genotypes that precisely pair mtDNAs from *Drosophila melanogaster* and *D. simulans* with nuclear genomes from wild‐type *D. melanogaster* (Montooth et al. 2010). Pairing the *D. simulans* mtDNA from the *simw* ^501^ strain with two *D. melanogaster* nuclear genomes reveals a strong mito‐nuclear epistatic interaction for fitness. The *D. simulans simw* ^501^ mtDNA is phenotypically wild type when combined with the *D. melanogaster AutW132* nuclear genome (hereinafter *Aut*), but is incompatible with the *D. melanogaster Oregon‐R* (hereinafter *OreR*) nuclear genome, resulting in a significant increase in development time and decrease in fitness of the (*simw* ^501^);*OreR* (mtDNA);nuclear genotype (Montooth et al. 2010; Meiklejohn et al. 2013). The molecular genetic basis of this interaction is an incompatible pairing between a single nucleotide polymorphism (SNP) in the *simw* ^501^ mitochondrial‐encoded tRNA^Tyr^ and a naturally segregating amino acid polymorphism present in the *OreR* nuclear‐encoded mitochondrial tyrosyl‐tRNA synthetase gene, *Aatm*. These mutations act epistatically to decrease OXPHOS activity, as predicted by the critical role that these genes play in mitochondrial protein translation (Meiklejohn et al. 2013). The four genotypes that we use here—(*ore*);*Aut*, (*ore*);*OreR*, (*simw* ^501^);*Aut*, and (*simw* ^501^);*OreR*—provide a well‐characterized model of epistasis between naturally occurring polymorphisms that affects energy metabolism and fitness, allowing us to test how internal and external environment influences the phenotypic expression of genetic interactions (Table 1). Fly cultures were maintained on Bloomington *Drosophila* Stock Center media with a 12:12h light/dark cycle, unless otherwise indicated.

**Table 1:** Biological interpretation of context‐dependent genetic effects in this study system

Interaction*	Tested in this system†	Biological interpretation
G × G	mtDNA × Nuclear	Phenotypic effects of mtDNA variation depend upon nuclear genomic variation (i.e., epistasis)
G × E	mtDNA | Nuclear × Photoperiod | T_DEV_ | T_MEASURE_	Phenotypic effects of variation in either genome depend upon the environment, which can also be interpreted as genetic variation for phenotypic plasticity
G × G × E	mtDNA × Nuclear × Photoperiod | T_DEV_ | T_MEASURE_	Phenotypic effects of the mitochondrial‐nuclear interaction are conditional on environment (i.e., context‐dependent epistasis)
G × E × E	mtDNA | Nuclear × Photoperiod × T_DEV_	The differential effect of photoperiod on the development time of particular genotypes depends upon development temperature
	mtDNA | Nuclear × T_MEASURE_ × T_DEV_	Phenotypic effects of variation in either genome on thermal plasticity of metabolic rate (the *Q*_10_) depend upon development temperature
G × G × E × E	mtDNA × Nuclear × Photoperiod × T_DEV_	Phenotypic effects of the mitochondrial‐nuclear interaction on development time in response to photoperiod are conditional upon development temperature
	mtDNA × Nuclear × T_MEASURE_ × T_DEV_	Phenotypic effects of the mitochondrial‐nuclear interaction on thermal plasticity of metabolic rate (the *Q*_10_) depend upon development temperature (as in Hoekstra et al. (2013))

^*^Phenotypic effects of any of these interactions may also differ between males and females (i.e., sex‐specific effects).

^†^“ | ” denotes one or the other factor.

### MANIPULATING DEVELOPMENTAL PHOTOPERIOD

We tested the specific prediction that an arrhythmic photoperiod (24:0h L/D, hereafter referred to as constant light) would accelerate growth rate in wild‐type genotypes, but that the increased energy demand of accelerated growth would induce a developmental delay of the incompatible (*simw* ^501^);*OreR* genotype at 16°C—a temperature where this genotype has a wild‐type development time (Hoekstra et al. 2013). We also tested whether constant light at 22°C would phenocopy the developmental delay caused by (*simw* ^501^);*OreR* at higher developmental temperatures (Hoekstra et al. 2013). We quantified the effect of extended day length on development time using four different combinations of temperature and light/dark cycle (16°C, 12:12 h; 16°C, 24:0 h; 22°C, 12:12 h; 22°C, 24:0 h). For each genotype, replicate pools of fifty 0–12 hour old eggs were collected into fresh food vials and randomly assigned to one of the four developmental treatments. We scored the number of new pupae and new adults to eclose once per day at 16°C and twice per day at 22°C for approximately 20 vials of each genotype under each developmental treatment. The fixed effects of genotype and photoperiod on development time within each temperature were tested using mixed‐model analysis of variance (ANOVA) models that were fit using restricted maximum likelihood and included rearing vial as a random factor. To provide context for interpreting the magnitude of the effect of constant light, we used developmental data from these same four genotypes at 25°C, 12:12 h from Hoekstra et al. (2013). All analyses were performed in the R statistical package (R Core Team 2013).

### ADULT MASS AND METABOLIC RATE

Incompatible (*simw* ^501^);*OreR* larvae have inefficient larval metabolism, manifest as higher metabolic rates and longer development times at 25°C, but develop and respire at a normal pace at 16°C (Hoekstra et al. 2013). To test whether this mito‐nuclear incompatibility also affects adult metabolism, we reared all four genotypes from egg to adult with controlled densities at 16°C or 25°C and measured mass and metabolic rate of 3–6 day old adults. Density was controlled by placing 50 eggs in each vial, with 20% more eggs for the incompatible genotype to account for its decreased hatch rate. At 48 hours post pupal eclosion, adult flies were lightly anaesthetized with CO_2_, sexed, and sorted into groups of 10 flies. The wet mass of each group of 10 flies was recorded to the nearest μg and adults were allowed to recover in fresh, yeasted food vials for at least 24 hours. Mass was log‐transformed to improve normality and genetic effects on mass were tested using ANOVA and Tukey's posthoc contrasts corrected for the number of multiple tests.

We used flow‐through respirometry to estimate routine metabolic rate (RMR, or hereafter, metabolic rate) as the volume of CO_2_ (VCO_2_) produced by groups of 10 female or male flies of the same genotype that were confined to a small, dark space to minimize activity. VCO_2_ is a good proxy for metabolic rate in insects like *D. melanogaster* that largely use carbohydrates for respiration and have a respiratory quotient of approximately one (Chadwick 1947). We measured at least 10 biological replicates of each combination of genotype, sex, development temperature (T_DEV_ = 16°C and 25°C), and measurement temperature (T_MEASURE_ = 16°C and 25°C) using offspring collected from multiple cultures and multiple parental generations to average across micro‐environmental effects. Metabolic rates were measured between 11:00 am and 7:00 pm, and genotypes were distributed across this timeframe and across respirometry chambers using a random, balanced design. All measurements were made in a Peltier‐controlled thermal cabinet (Tritech Research, Inc.), and measurement temperature was monitored using a thermocouple meter wired into an empty respirometry chamber.

For flow‐through measurement of adult VCO_2_, we pushed CO_2_‐free air through glass respirometry chambers containing flies at a rate of 100 mL/min. Air that leaves the chamber carries CO_2_ produced by the flies, as well as water. The water vapor was removed from the airstream using magnesium perchlorate, and the CO_2_ in the airstream was measured using a Licor 7000 infrared CO_2_ detector (Licor, Lincoln, NE). We used the RM8 Intelligent Multiplexer to switch the airstream sequentially through five respiratory chambers (Sable Systems International, Las Vegas, NV), one of which serves as an empty baseline chamber. Each experimental run measured the VCO_2_ of four pools of 10 adult flies, each sampled twice for 10 minutes during a 100‐minute period. There was no death from this treatment.

Baseline CO_2_ values were recorded before and after each sample and used to drift‐correct CO_2_‐tracings using the two‐endpoint automatic method in Expedata, version 1.1.15 (Sable Systems International, Las Vegas, NV). Raw CO_2_ values were converted from parts per million to μL/h (VCO_2_) and then log‐transformed to improve normality and homoscedasticity. To allow metabolic rate to acclimate in response to temperature shifts (e.g., T_DEV_ = 16°C and T_MEASURE_ = 25°C), we used the second recording of each respirometry run to estimate metabolic rate such that flies were acclimated for 50–80 min.

Because there is measurement error in adult body mass, we estimated the scaling relationship between mass and VCO_2_ using Type II Model regression implemented with smatR, version 3.4.3 (Warton et al. 2006). When justified by a homogeneity of slopes test for the log–log relationship between mass and VCO_2_, we fit a common slope to all genotypes and tested for shifts along the common x axis (i.e., differences in mass) and for shifts in elevation (i.e., differences in mass‐specific metabolic rate) among genotypes. Across genotypes within each of the four T_MEASURE_ × Sex combinations, we were able to fit a common slope and test for genotype differences in mass and in mass‐specific metabolic rate. We could then correct for the effect of mass on metabolic rate by taking the residuals of each of these regressions and adding back the grand mean of all fitted values to provide meaningful scale. We refer to these values as mass‐corrected metabolic rates. We tested for the fixed effects of T_DEV_, mtDNA, nuclear genotype, and all possible interactions on mass‐corrected metabolic rate using analysis of variance (ANOVA).

Respirometry chambers were housed inside infrared activity detectors (AD‐2, Sable Systems International, Las Vegas, NV), providing a simultaneous measurement of activity. We summarized the activity data by taking the median absolute difference of activity across each seven‐minute metabolic rate measurement. This measure of activity neither significantly affected metabolic rate nor interacted with any genetic effects and was not included in our final statistical models.

### ADULT REPRODUCTIVE TRAITS

Incompatible (*simw* ^501^);*OreR* females have significantly reduced fecundity, measured as the number of eggs laid over 10 days (Meiklejohn et al. 2013). To test whether males of this genotype also suffer a decrease in reproductive fitness, we measured one aspect of male fertility—the number of offspring sired by an individual male mated to virgin females of a control wild‐type genotype, *Canton‐S*. Thirty males were assayed for fertility across two experimental blocks that spanned multiple parental generations and used slightly different female genotypes. Both female genotypes were *Canton‐S*, but in the first block the strain carried the *cn,bw* eye mutation. Block was included as a fixed factor in the analysis. However, rank orders of genotypes for the number of females fertilized and the number of offspring sired were similar between blocks.

Males were given 48 hours to mate with three virgin females. After 48 hours, we placed each female into a separate vial to lay fertilized eggs for an additional week. Progeny emerging from each vial were counted every other day until all progeny were counted. Males that sired fewer than 25 offspring were removed from the analysis, which resulted in a sample size of 29 males per genotype, except for (*simw* ^501^);*Aut* for which *n* = 28 males. This outlier removal essentially removed the occasional sterile male, which were few and evenly distributed (1–2 males) across genotypes. By placing females in separate vials after they were housed with the focal male, we could infer how many females were mated by each male, with the caveat that some females may have laid eggs in the first vial, but not in their individual vials. The median number of females that produced offspring in their vial per male was 3 (mean = 2.64) and this was not affected by genotype (*P* > 0.15 for genotype and all interactions). We estimated fertility as the total progeny sired by each male divided by the number of females with whom that male produced progeny. We tested for fixed effects of mtDNA, nuclear genotype, experimental block, and the interaction between these factors using ANOVA.

## Results

### CONSTANT LIGHT PHENOCOPIES THE TEMPERATURE‐DEPENDENT DEVELOPMENTAL DELAY OF A MITO‐NUCLEAR INCOMPATIBILITY

**Figure 1:**
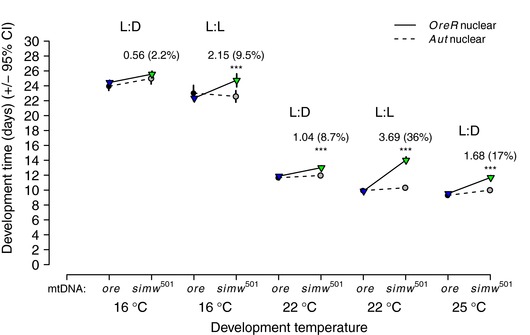
An arrhythmic photoperiod phenocopies the effects of increasing temperature on a mito‐nuclear incompatibility that extends development. An arrhythmic, constant‐light photoperiod (L:L) accelerates development in control genotypes relative to a rhythmic photoperiod (L:D), but delays development in the mito‐nuclear incompatible genotype (*simw* ^501^);*OreR* (Table S1). For comparison, the developmental delay of (*simw* ^501^);*OreR* larvae relative to control genotypes under constant light at 22°C is greater than the delay observed at 25°C under fluctuating light (L:D) (25°C data from Hoekstra et al. (2013)). Asterisks denote a significant effect of the mtDNA × nuclear genetic interaction within each temperature‐photoperiod combination at the level of *P* < 0.0001 (Table S2), with the associated mean days delayed of (*simw* ^501^);*OreR* relative to (*simw* ^501^);*Aut* and the percent increase in development time in parenthesis.

The developmental delay of incompatible (*simw* ^501^);*OreR* larvae is strongly mediated by temperature, with warmer temperatures exacerbating and cooler temperatures masking the delay (Hoekstra et al. 2013). *Drosophila* development rate depends on variation in the rhythmicity of the photoperiod (e.g., Kyriacou et al. 1990; Paranjpe et al. 2005). We tested whether an arrhythmic, constant‐light photoperiod (24:0h L:D) that typically accelerates larval‐to‐adult development (Paranjpe et al. 2005) could phenocopy this developmental delay in a manner similar to the accelerating effect of increased temperature. The accelerating effect of constant light influenced the severity of the (*simw* ^501^);*OreR* developmental delay in a pattern remarkably similar to the effect of increased development temperature (Fig. 1, Tables S1 and S2). The incompatible (*simw* ^501^);*OreR* genotype developed at the same pace as other genotypes when reared at 16°C with a fluctuating photoperiod (12:12h L:D) (mtDNA × Nuclear: *F*_1, 80_ = 0.38, *P* = 0.5413). However, the incompatible (*simw* ^501^);*OreR* genotype experienced a significant developmental delay relative to other genotypes when developed at 16°C with constant light (24:0h L:D) (mtDNA × Nuclear: *F*_1, 79_ = 30.13, *P* <0.0001). At 22°C, where the incompatible genotype normally experiences a significant developmental delay with a fluctuating photoperiod, the constant light photoperiod magnified the developmental delay (Photoperiod × mtDNA × Nuclear: *F*_1, 153_ = 299.07, *P* <0.0001). This was a particularly striking effect; constant light accelerated development of compatible mito‐nuclear genotypes by ∼2 days at 22°C, which is comparable to development time at 25°C. In contrast, constant light at 22°C significantly slowed development of the incompatible (*simw* ^501^);*OreR* genotype relative to other genotypes and relative to its own development time at 22°C under fluctuating light. This resulted in an ∼4‐day developmental delay between (*simw* ^501^);*OreR* and compatible genotypes (Fig. 1). Within each developmental temperature, the magnitude of the effect of the mito‐nuclear genetic interaction on development time was conditional on photoperiod (G × G × E, Table 1; *P* < 0.0001, Table S1). However, there was no evidence that the highest order interaction between development temperature, photoperiod, mtDNA, and nuclear genotype affected development time (G × G × E × E, Table 1; *F*_1,312_ = 0.41, *P* = 0.5244).

### THE MITO‐NUCLEAR INCOMPATIBILITY DOES NOT AFFECT ADULT MASS

Gene‐environment (G × E) effects on adult mass were dominated by interactions between nuclear genotype and the known effects of both development temperature and sex on mass (Supplemental Table S3). Relative to these large effects, there was a small, but statistically significant effect of the mito‐nuclear interaction on mass (mtDNA x nuclear: *F*_1,317_ = 6.078, *P* = 0.0142). Rather than eclosing as smaller adults, (*simw* ^501^);*OreR* adults were slightly larger than (*ore*);*OreR* adults (*P*_Tukey_ = 0.04). However, the magnitude of the effect was small (+0.04 mg/10 flies) and the mtDNA × nuclear interaction was only statistically significant for females raised at 16°C (Table S4). In summary, adults with a mito‐nuclear incompatibility that survived development achieved body masses that were similar to or greater than compatible genotypes.

### ADULT METABOLIC RATE IS MORE ROBUST TO MITO‐NUCLEAR INTERACTIONS THAN IS LARVAL METABOLIC RATE

The scaling of metabolic rate as a function of mass can be characterized by the slope of the relationship between *ln*(metabolic rate) and *ln*(mass) (i.e., the mass‐scaling exponent). The mass‐scaling exponent differed slightly, but significantly between sexes and between measurement temperatures for adults in this study (*P* < 0.0001 for both Sex × Mass and T_MEASURE_ × Mass interactions). However, within each combination of sex and T_MEASURE_, there was no evidence that genotype or development temperature affected the mass‐scaling exponent (*P* > 0.05 for both). Thus, we fit common slopes to the metabolic rate data for all genotypes within each sex‐T_MEASURE_ combination to test for effects of mito‐nuclear genotype (Fig. S1 and Table S5). Within each sex‐T_MEASURE_ combination, the range of masses for all genotypes were largely overlapping and there was no evidence for differences in mass among genotypes (Fig. S1 and Table S5).

**Figure 2:**
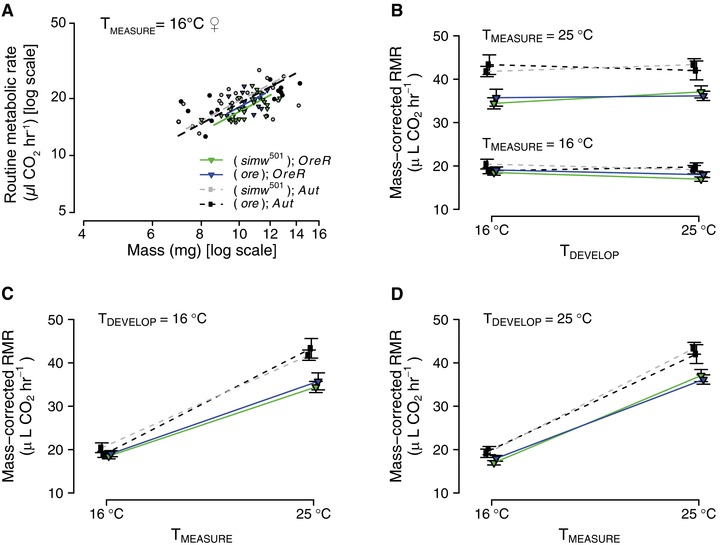
Female adult metabolic rate is robust to mito‐nuclear genetic effects. (A) The only mito‐nuclear genetic effect was a small, but significant decrease in 16°C routine metabolic rate (RMR) in incompatible (*simw* ^501^);*OreR* females (*P* < 0.05, Table S5). (B) At each measurement temperature, mass‐corrected metabolic rates were similar for females developed at different temperatures, indicating strong physiological homeostasis for metabolic rate. (C and D) Thermal reaction norms show that the *Q*_10_ for female mass‐corrected metabolic rate is similar under both developmental temperatures, and that incompatible (*simw* ^501^);*OreR* females have similar metabolic plasticity as their nuclear genotypic control (*ore*);*OreR*. The analogous data for adult males are provided in Figure S2 and revealed similar patterns as observed in females. The mtDNA × nuclear interaction did not affect mass‐corrected metabolic rate of males or females at either measurement temperature (*P* > 0.28; Table S6). Error bars are ±1 SEM and symbols and colors designate genotypes as indicated in panel A.

In contrast to the larval life stage where (*simw* ^501^);*OreR* larvae have significantly elevated 25°C metabolic rates (Hoekstra et al. 2013), there was no evidence of elevated metabolic rate in the incompatible (*simw* ^501^);*OreR* adults measured at any temperature. The only significant effect of the mito‐nuclear genotype on adult metabolic rate was a lower (*simw* ^501^);*OreR* metabolic rate relative to compatible genotypes at 16°C. However, once again, this effect was small and only in females (Fig. 2A, Fig. S1 and Table S5). Male metabolic rate was unaffected by mito‐nuclear genotype (Fig. S1 and Table S5). Significant effects of the nuclear genome at 25°C (Table S5) and in our prior work (Hoekstra and Montooth 2013; Hoekstra et al. 2013; Greenlee et al. 2014) demonstrate the sensitivity of this method to detect both genetic and environmental effects on metabolic rate.

Mass‐corrected metabolic rates allow for comparisons of genotypes across development and measurement temperatures (i.e., metabolic plasticity) (Fig. 2B–D, Fig. S2, Tables S6 and S7). This analysis revealed that the lower 16°C metabolic rates in (*simw* ^501^);*OreR* females were largely the consequence of lower metabolic rates of females developed at 25°C and measured at 16°C. However, this difference was not statistically significant and supports generally weak effects of this genetic interaction on adult, relative to larval, metabolic rates. There was no evidence that the mito‐nuclear interaction affected adult male mass‐corrected metabolic rate within any combination of development or measurement temperatures (Fig. S2 and Table S6). In contrast to larvae, where (*simw* ^501^);*OreR* have compromised thermal plasticity of metabolic rate (i.e., the *Q*_10_ for metabolic rate), we found no evidence that adults of this genotype differed in their *Q*_10_ for metabolic rate (T_MEASURE_ × mtDNA × nuclear, *P* > 0.65 for both sexes) and this was independent of development temperature (T_MEASURE_ × T_DEV_ × mtDNA × nuclear, *P* > 0.50 for both sexes) (Table S7). Thus, relative to larvae, both adult metabolic rate and metabolic plasticity were more robust to the effects of this mito‐nuclear genetic incompatibility.

### MITO‐NUCLEAR EFFECTS ON REPRODUCTIVE FITNESS ARE STRONGER IN FEMALES

**Figure 3:**
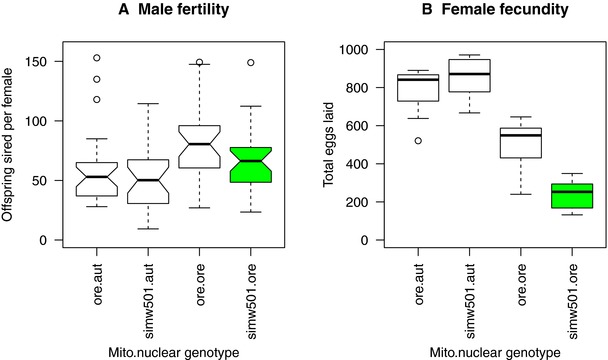
Mito‐nuclear effects on reproductive phenotypes are stronger in females. (A) Male fertility was not significantly affected by the mito‐nuclear incompatibility (mtDNA × nuclear: *F*_1, 107_ = 0.185, *P* = 0.668). (B) This was in contrast to strong effects of mito‐nuclear genotype on female fecundity (mtDNA × nuclear: *F*_1, 31_ = 16.976, *P* = 0.0003), with females of the incompatible (*simw* ^501^);*OreR* genotype laying on average 48% the number of eggs produced by (*ore*);*OreR* females. Female fecundity data from Meiklejohn et al. (2013) are total eggs produced over 10 days by individual females for *n* = 7–10 females per genotype. Whiskers on boxplots represent interquartile ranges. Green denotes the mitochondrial‐nuclear incompatible genotype, as in other figures.

Patterns of male fertility and female fecundity provided further evidence that the effects of the mito‐nuclear incompatibility were sex specific. The mito‐nuclear interaction did not compromise the number of offspring sired by males (mtDNA × nuclear: *F*_1, 107_ = 0.185, *P* = 0.668) (Fig. 3A, Table S8). This was in contrast to strong mito‐nuclear effects on female fecundity, with females of the (*simw* ^501^);*OreR* genotype producing ∼50% fewer eggs than (*ore*);*OreR* females (Fig. 3B, Table S8) (Meiklejohn et al. 2013). Thus, while both sexes generally maintain metabolic rate independent of mito‐nuclear genotype, (*simw* ^501^);*OreR* females appear to do so at the cost of egg production.

## Discussion

Previously, we found that a synergistic, epistatic interaction between polymorphisms in the mt‐tRNA^Tyr^ and the nuclear‐encoded, mitochondrially targeted tRNA‐synthetase for this mt‐tRNA decreases OXPHOS activity and negatively impacts a number of life‐history traits in a temperature‐dependent manner in *Drosophila* (Montooth et al. 2010; Meiklejohn et al. 2013, Hoekstra et al. 2013; Zhang et al. 2017). Here, we show that the phenotypic expression of this genetic interaction is generally environment dependent and depends on intrinsic factors such as life stage and sex in a manner consistent with greater energy demands revealing fitness effects of mutations that compromise metabolism.

### THE PHYSIOLOGICAL BASIS OF ENVIRONMENT‐DEPENDENT, MITO‐NUCLEAR EFFECTS

Many organismal traits, particularly in ectotherms, are temperature dependent, including the universal, but mechanistically not well understood, unimodal thermal performance curve for metabolic rate (Angiletta 2009; Schulte 2015; DeLong et al. 2017). Given this relationship between temperature and metabolic processes, the thermal environment is likely an ecologically relevant and critical determinant of the relationship between genotype and phenotype for traits that depend on metabolic function. Phenotypic effects of cytonuclear interactions, including the mito‐nuclear incompatibility described here, can be temperature sensitive (Arnquvist et al. 2010; Dowling et al. 2007a; Hoekstra et al. 2013). The temperature dependence of these mito‐nuclear genetic effects could result from direct thermodynamic effects on the physical interaction between the mutations, for example between mutations in the mt‐tRNA^Tyr^ and its tRNA synthetase. Alternatively, the temperature dependence may arise indirectly, as metabolic rate and development rate increase with temperature and place greater demand on the energetic products of mito‐nuclear interactions. This latter, energy‐dependent explanation is also consistent with the observations that mitochondrial genetic effects can be temperature sensitive (Pichaud et al. 2013) and both mitochondrial and mito‐nuclear effects can be diet sensitive (Zhu et al. 2014; Ballard and Youngson 2015; Mossman et al. 2016). Here, we found that manipulating the photoperiod to accelerate growth rate independent of temperature produced patterns of context‐dependent mito‐nuclear genetic effects strikingly similar to those revealed by varying development temperature. While this does not exclude the possibility that there are direct thermal effects on the physical interaction between mt‐tRNA and tRNA synthetase, it demonstrates that temperature is not required to expose this genetic interaction and suggests a more general physiological explanation; external and internal contexts that place greater demand on energy metabolism expose deleterious effects of genetic interactions that compromise energy supply.

In some insects, including *Drosophila*, pupal eclosion behavior is under control of the circadian clock (Kyriacou et al. 1990; Paranjpe et al. 2005). Under cyclic or rhythmic photoperiodic regimes, the circadian clock entrains to the light cycle and eclosion behavior is gated such that pupae will delay the initiation of eclosion behavior until lights off in order to synchronize eclosion with dawn. In arrhythmic photoperiodic environments, however, eclosion behavior is unregulated and pupae initiate eclosion behavior as quickly as possible. While compatible mito‐nuclear genotypes developed under a constant‐light, arrhythmic photoperiod experienced an unregulated acceleration of development, incompatible mito‐nuclear genotypes do not appear to have the energetic capacity to similarly accelerate growth. (*simw* ^501^);*OreR* larvae also have significantly reduced thermal plasticity for metabolic rate (i.e., they have a very low *Q*_10_) (Hoekstra et al. 2013). Thus under two independent contexts that normally accelerate growth (constant light and warm temperatures), this mito‐nuclear incompatibility appears to limit larval growth. Mito‐nuclear incompatibilities likely limit the scope for growth, potentially because incompatibilities compromise ATP production and result in energy supplies that are very close to the increased energy demands of rapid larval growth.

### ONTOGENY OF THE ENERGY BUDGET

Even at temperatures that most exacerbate mito‐nuclear effects on larval metabolic rate and survivorship (Hoekstra et al. 2013), we found that the effects of this mito‐nuclear incompatibility on adult metabolic rate are minimal. This suggests that the deleterious effects of mito‐nuclear incompatibility on metabolic rate and survivorship may be alleviated by the cessation of growth. Larval growth in *D. melanogaster* proceeds extremely quickly and challenges metabolic processes (Church and Robertson 1966; Tennessen et al. 2011). Furthermore, metabolism during growth is estimated to be 40–79% above that of fully developed conspecifics (Parry 1983), and cessation of growth in holometabolous insects is correlated with an ontogenetic decrease in the mass‐scaling exponent relating metabolic rate to mass (Glazier 2005; Callier and Nijhout 2012; Greenlee et al. 2014; Maino and Kearney 2014). Thus, the cessation of growth in *Drosophila* likely results in the excess metabolic capacity needed to compensate adult metabolic rate of incompatible, mito‐nuclear genotypes. Hoekstra et al. (2013) also observed that for those individuals that survive to pupation, there is no further effect of the mito‐nuclear incompatibility on survival through metamorphosis, when metabolic rates decrease to a minimum (Dobzhansky and Poulson 1935; Merkey et al. 2011) and those individuals that have committed to pupation appear to have the energy stores needed for successful metamorphosis. Consistent with this, we observed that adult (*simw* ^501^);*OreR* attain similar mass at eclosion as do adults with compatible mitochondrial and nuclear genomes. Changes in the balance of energy supply and demand of holometabolous insects across development (e.g., Merkey et al. 2011) likely generate important ontogenetic contingency for the fitness effects of mutations that affect metabolism. The fate of conditionally neutral alleles, such as those underlying this mito‐nuclear incompatibility, will then depend not only on what environments are experienced, but when those environments are experienced in an organism's lifespan (*sensu* Diggle 1994).

### SEX‐SPECIFIC COSTS OF REPRODUCTION

The small effect that this mito‐nuclear interaction did have on adult metabolic rate was sex specific. (*simw* ^501^);*OreR* females had significantly depressed metabolic rates when measured at cool temperatures. There was some indication that this was driven by disruption of metabolic plasticity, similar to what we have observed in larvae of this genotype, although of much weaker effect; (*simw* ^501^);*OreR* females developed at one temperature and measured at the other temperature had the lowest mass‐corrected metabolic rates relative to all other compatible genotypes.

If energy stores in mito‐nuclear incompatible individuals are limited, then the maintenance of metabolic rate using an inefficient OXPHOS system might support adult maintenance, but at a cost to more energetically demanding function, such as reproduction. We observed this pattern in females, but not in males. Females with incompatible mito‐nuclear genomes laid far fewer eggs, while the fertility of males with the same mito‐nuclear combination was unaffected. The fecundity defects in (*simw* ^501^);*OreR* females are also strongly temperature dependent and involve defects in the development and maintenance of the ovary (Zhang et al. 2017). Furthermore, mothers of this genotype developed at 28°C produce defective eggs that have a lower probability of being fertilized and, when fertilized, die during embryogenesis presumably due to insufficient maternal provisioning or the inheritance of subfunctional mitochondria (Zhang et al. 2017).

Although we measured only one aspect of reproductive fitness for each sex, this pattern is consistent with a higher cost of reproduction in females (Bateman 1948) and with empirical estimates of the costs of gamete production that suggest that high costs of egg production may specifically constrain female gamete production (Hayward and Gillooly 2011). Oogenesis in *Drosophila* is regulated in response to nutrient availability (Drummond‐Barbosa and Spradling 2001), but nutrient checkpoints for spermatogenesis are less well studied (but see e.g., McLeod et al. 2010; Yang and Yamashita 2015). Further experiments are warranted to determine whether there are more subtle fertility defects in (*simw* ^501^);*OreR* males, as cytoplasmic effects on sperm morphology and viability have been measured in seed beetles (Dowling et al. 2007b). However, these viability differences do not contribute to cytoplasmic effects on sperm competition in seed beetles or in *D. melanogaster* (Dowling et al. 2007c; Friberg and Dowling 2008). Our findings suggest that homeostasis for metabolic rate (or ATP production) combined with potentially differential costs of gametogenesis, may generate sex‐specific allocation tradeoffs between maintenance and reproduction as a consequence of genetic variation in metabolic processes.

## Conclusion

Our observation that the degree of expression of a mito‐nuclear incompatibility correlates with energetic demand—among developmental treatments that accelerate growth rate, across developmental stages with substantial differences in the cost of growth, and between sexes with putatively different costs of reproduction—suggests that the phenotypic effects of genetic interactions that impact metabolism may depend broadly on the context of energy use and the metabolic cost of producing focal traits. The balance between energy supply and demand may change as a function of the external (e.g., temperature or photoperiod) or internal (e.g., life stage, sex, or tissue) environment in which a trait is expressed. Genetic variants that compromises energy supply—via nutrient acquisition, storage, or conversion to ATP—may then manifest as phenotypic effects on performance and fitness under conditions where demand is high, but be masked under conditions where supplies are in excess of lower demands. The context‐dependent genotype‐phenotype relationships that we describe exemplify how energy allocation principles and design constraints can generate complicated environment‐dependence, potentially confounding attempts to define fitness in energetic terms (e.g., Bruning et al. 2013). Yet, many components of fitness are expected to depend on the pathways of metabolism, and the variable phenotypic expression of mutations in these pathways presents a dynamic and perhaps challenging context for both purifying and adaptive selection, as context‐dependent mutational effects only experience selection in a fraction of possible environments (Van Dyken and Wade 2010). It is important to consider that the genetic variants used in this study are from different species and are isolated in an inbred genetic background, which may not represent the allelic or allelic‐by‐environment effects of these variants in an outbred genetic background (Mueller and Cabral 2012). These constructed genotypes demonstrate the potential for condition‐dependent effects of mito‐nuclear interactions on metabolism and fitness. Nonetheless, both the nuclear and mitochondrial variants studied here exist in natural populations of fruit flies (Meiklejohn et al. 2013) and may contribute to natural variation in metabolism. Our findings motivate further investigation—including in an outbred genetic context—of the role of energy demand in mediating the phenotypic effects of genetic variation in metabolism, mito‐nuclear or otherwise, as this will have significant implications for the persistence of genetic variation for metabolism and the evolution of metabolic performance in natural populations.

Associate Editor: Z. Gompert

## Supporting information


**Table S1**. Temperature‐dependent effects of mito‐nuclear interactions on development time are modified by the developmental photoperiod.
**Table S2**. Developmental temperature and photoperiod can both independently modulate mito‐nuclear genetic effects on development time.
**Table S3**. Gene‐environment interactions affect adult body mass.
**Table S4**. Mito‐nuclear genetic effects on adult body mass are specific to females developed at 16°C.
**Table S5**. Mito‐nuclear genetic effects on adult metabolic rate are specific to females developed at 16°C.
**Table S6**. Mito‐nuclear interactions do not affect adult mass‐corrected metabolic rate.
**Table S7**. Mito‐nuclear interactions do not affect metabolic plasticity (i.e., the *Q*_10_ for metabolic rate) in adult females or males.
**Table S8**. Mito‐nuclear interactions affect female, but not male, reproductive fitness.
**Figure S1**. Weak effects of mito‐nuclear genotype on adult metabolic rate depend upon sex and measurement temperature.
**Figure S2**. Adult male metabolic plasticity is not affected by mito‐nuclear genetic effects.

## References

[evl347-bib-0001] Agrawal, A. A. , J. K. Conner , and S. Rasmann . 2010 Tradeoffs and adaptive negative correlations in evolutionary ecology *In* Bell M., Eanes W., Futuyma D, and Levinton J., eds. Evolution After Darwin: the First 150 Years. Sinauer, Sunderland, Massachusetts.

[evl347-bib-0002] Angilletta, M. Jr . 2009 Thermal adaptation: A theoretical and empirical synthesis. Oxford Univ. Press, Oxford, UK

[evl347-bib-0003] Arnqvist, G. , D. K. Dowling , P. Eady , L. Gay , T. Tregenza , M. Tuda et al. 2010 Genetic Architecture of metabolic rate: environment specific epistasis between mitochondrial and nuclear genes in an insect. Evolution 64:3354–3363. 20874734 10.1111/j.1558-5646.2010.01135.x

[evl347-bib-0060] Ashmore, L. J. , and A. Sehgal . 2003 A Fly's eye view of circadian entrainment. J. Biol. Rhythms. 18:206–216. 12828278 10.1177/0748730403018003003

[evl347-bib-0004] Asplen, M. K. , E. Bruns , A. S. David , R. F. Denison , B. Epstein , M. C. Kaiser et al. 2012 Do trade‐offs have explanatory power for the evolution of organismal interactions? Evolution 66:1297–1307. 22519772 10.1111/j.1558-5646.2011.01573.x

[evl347-bib-0005] Ballard, J. W. O. , and N. A. Youngson . 2015 Review: can diet influence the selective advantage of mitochondrial DNA haplotypes? Biosc. Rep. 35:e00277. 10.1042/BSR20150232 PMC4708006 26543031

[evl347-bib-0006] Bateman, A. J. 1948 Intra‐sexual selection in *Drosophila* . Heredity 2:349–368. 18103134 10.1038/hdy.1948.21

[evl347-bib-0007] Bruning, A. , J. D. Gaitán‐Espitia , A. González , J. L. Bartheld , and R. F. Nespolo . 2013 Metabolism, growth, and the energetic definition of fitness: a quantitative genetic study in the land snail *Cornu aspersum* . Physiol. Biochem. Zool. 86:538–546. 23995484 10.1086/672092

[evl347-bib-0061] Burton, R. S. , and F. S. Barreto . 2012 A disproportionate role for mtDNA in Dobzhansky. Muller incompatibilities? Mol. Ecol. 21:4942–4957. 22994153 10.1111/mec.12006

[evl347-bib-0008] Callier, V. , and H. F. Nijhout . 2012 Supply‐side constraints are insufficient to explain the ontogenetic scaling of metabolic rate in the tobacco hornworm, *Manduca sexta* . PLoS ONE 7:e45455. 23029018 10.1371/journal.pone.0045455 PMC3446882

[evl347-bib-0009] Chadwick, L. E . 1947 The respiratory quotient of *Drosophila* in flight. Biol. Bull. 93:229–239. 18919572

[evl347-bib-0010] Chandler, C. H. , S. Chari , and I. Dworkin . 2013 Does your gene need a background check? How genetic background impacts the analysis of mutations, genes, and evolution. Trends Genet. 29:358–366. 23453263 10.1016/j.tig.2013.01.009 PMC3692003

[evl347-bib-0011] Church, R. B. , and F. W. Robertson . 1966 A biochemical study of the growth of *Drosophila melanogaster* . J. Exp. Zool. 162:337–351.

[evl347-bib-0012] Clarke, A. , and K. P. P. Fraser . 2004 Why does metabolism scale with temperature? Funct. Ecol. 18:243–251.

[evl347-bib-0013] DeLong, J. P. , J. P. Gibert , T. M. Luhring , G. Bachman , B. Reed et al. 2017 The combined effects of reactant kinetics and enzyme stability explain the temperature dependence of metabolic rates. Ecol. Evol. 7:3940–3950. 28616189 10.1002/ece3.2955 PMC5468145

[evl347-bib-0014] Diggle, P. K. 1994 The expression of andromonoecy in *Solanum‐Hirtum* (*Solanaceae*)—phenotypic plasticity and ontogenic contingency. Am. J. Bot. 81:1354–1365.

[evl347-bib-0015] Dobzhansky, T. , and D. F. Poulson . 1935 Oxygen consumption of *Drosophila* pupae. Z. Vergl. Physiol. 22:473–478.

[evl347-bib-0016] Dowling, D. K. , K. C. Abiega , and G. Arnqvist . 2007a Temperature‐specific outcomes of cytoplasmic‐nuclear interactions on egg‐to‐adult development time in seed beetles. Evolution 61:194–201. 17300438 10.1111/j.1558-5646.2007.00016.x

[evl347-bib-0017] Dowling, D. K. , A. L. Nowostawski , and G. Arnqvist . 2007b Effects of cytoplasmic genes on sperm viability and sperm morphology in a seed beetle: implications for sperm competition theory? J. Evol. Biol. 20:358–368. 17210029 10.1111/j.1420-9101.2006.01189.x

[evl347-bib-0018] Dowling, D. K. , U. Friberg , and G. Arnqvist . 2007c A comparison of nuclear and cytoplasmic genetic effects on sperm competitiveness and female remating in a seed beetle. J. Evol. Biol. 20:2113–2125. 17956382 10.1111/j.1420-9101.2007.01433.x

[evl347-bib-0019] Drummond‐Barbosa, D. , and A. C. Spradling . 2001 Stem cells and their progeny respond to nutritional changes during *Drosophila* oogenesis. Dev. Biol. 231:265–278. 11180967 10.1006/dbio.2000.0135

[evl347-bib-0020] Flatt T., and Heyland A., eds. 2011 Mechanisms of life history evolution: The genetics and physiology of life history traits and trade‐offs. Oxford Univ. Press, Oxford, U. K.

[evl347-bib-0021] Friberg, U. , and D. K. Dowling . 2008 No evidence of mitochondrial genetic variation for sperm competition within a population of *Drosophila melanogaster* . J. Evol. Biol. 21:1798–1807. 18643860 10.1111/j.1420-9101.2008.01581.x

[evl347-bib-0022] Glazier, D. S. 2005 Beyond the ‘3/4‐power law’: variation in the intra‐ and interspecific scaling of metabolic rate in animals. Biol. Rev. 80:611–662. 16221332 10.1017/S1464793105006834

[evl347-bib-0023] Greenlee, K. J. , K. L. Montooth , and B. R. Helm . 2014 Predicting performance and plasticity in the development of respiratory structures and metabolic systems. Integr. Comp. Biol. 54:307–322. 24812329 10.1093/icb/icu018 PMC4097113

[evl347-bib-0024] Harshman, L. G. , and A. J. Zera . 2007 The cost of reproduction: the devil in the details. TREE 22:80–86. 17056152 10.1016/j.tree.2006.10.008

[evl347-bib-0025] Hartman, J. L. , B. Garvik , and L. Hartwell . 2001 Principles for the buffering of genetic variation. Science 291:1001–1004. 11232561 10.1126/science.1056072

[evl347-bib-0026] Hayward, A. , and J. F. Gillooly . 2011 The cost of sex: quantifying energetic investment in gamete production by males and females. PLoS ONE 6:e16557. 21283632 10.1371/journal.pone.0016557 PMC3026017

[evl347-bib-0027] Hoekstra, L. A. , and K. L. Montooth . 2013 Inducing extra copies of the *Hsp70* gene in *Drosophila melanogaster* increases energetic demand. BMC Evol. Biol. 13:68. 23510136 10.1186/1471-2148-13-68 PMC3641968

[evl347-bib-0028] Hoekstra, L. A. , M. A. Siddiq , and K. L. Montooth . 2013 Pleiotropic effects of a mitochondrial‐nuclear incompatibility depend upon the accelerating effect of temperature in *Drosophila* . Genetics 195:1129–1139. 24026098 10.1534/genetics.113.154914 PMC3813842

[evl347-bib-0029] Holmbeck, M. A. , J. R. Donner , E. Villa‐Cuesta , and D. M. Rand . 2015 A *Drosophila* model for mito‐nuclear diseases generated by an incompatible interaction between tRNA and tRNA synthetase. Dis. Models Mechan. 8:843–854. 10.1242/dmm.019323 PMC4527286 26035388

[evl347-bib-0030] Kammenga, J. E. 2017 The background puzzle: how identical mutations in the same gene lead to different disease symptoms. FEBS J. 284:3362–3373 28390082 10.1111/febs.14080

[evl347-bib-0031] Kondrashov, A. S. , and D. Houle . 1994 Genotype‐environment interactions and the estimation of the genomic mutation rate in *Drosophila melanogaster* . Proc. Roy Soc. B 258:221–227. 10.1098/rspb.1994.0166 7886063

[evl347-bib-0032] Kyriacou, C. P. , M. Oldroyd , J. Wood , M. Sharp , and M. Hill . 1990 Clock mutations alter developmental timing in *Drosophil*a. Heredity 64:395–401. 2113515 10.1038/hdy.1990.50

[evl347-bib-0033] Lachance, J. , L. Jung , and J. R. True . 2013 Genetic background and G × E interactions modulate the penetrance of a naturally occurring wing mutation in *Drosophila melanogaster* . G3 3:1893–1901. 24002866 10.1534/g3.113.007831 PMC3815054

[evl347-bib-0034] Leopold, P. , and N. Perrimon . 2007 *Drosophila* and the genetics of the internal milieu. Nature 450:186–188. 17994083 10.1038/nature06286

[evl347-bib-0035] Mackay, T. F. C. 2014 Epistasis and quantitative traits: using model organisms to study gene‐gene interactions. Nat. Rev. Genet. 15:22–33. 24296533 10.1038/nrg3627 PMC3918431

[evl347-bib-0036] Maino, J. L. , and M. R. Kearney . 2014 Ontogenetic and interspecific metabolic scaling in insects. Am. Nat. 184:695–701. 25438170 10.1086/678401

[evl347-bib-0037] McLeod, C. J. , L. Wang , C. Wong , and D. L. Jones . 2010 Stem cell dynamics in response to nutrient availability. Curr. Biol. 2:2100–2105. 10.1016/j.cub.2010.10.038 PMC3005562 21055942

[evl347-bib-0038] Meiklejohn, C. D. , M. A. Holmbeck , M. A. Siddiq , D. N. Abt , D. M. Rand et al. 2013 An incompatibility between a mitochondrial tRNA and its nuclear‐encoded tRNA synthetase compromises development and fitness in *Drosophila* . PLoS Genet. 9:e1003238. 23382693 10.1371/journal.pgen.1003238 PMC3561102

[evl347-bib-0039] Merkey, A. B. , C. K. Wong , D. K. Hoshizaki , and A. G. Gibbs . 2011 Energetics of metamorphosis in *Drosophila melanogaster* . J. Insect Physiol. 57:1437–1445. 21810426 10.1016/j.jinsphys.2011.07.013

[evl347-bib-0040] Montooth, K. L. , C. D. Meiklejohn , D. N. Abt , and D. M. Rand . 2010 Mitochondrial‐nuclear epistasis affects fitness within species but does not contribute to fixed incompatibilities between species of *Drosophila* . Evolution 64: 3364–3379. 20624176 10.1111/j.1558-5646.2010.01077.x PMC2997886

[evl347-bib-0041] Mossman, J. A. , L. M. Biancani , C‐T. Zhu , and D. M. Rand . 2016 Mitonuclear epistasis for development time and its modification by diet in *Drosophila* . Genetics 203:463–484. 26966258 10.1534/genetics.116.187286 PMC4858792

[evl347-bib-0064] Mueller, L. D. , and L. G. Cabral . 2012 Does phenotypic plasticity for adult size versus food level in Drosophila melanogaster evolve in response to adaptation to different rearing densities? Evolution 66:263–271. 22220880 10.1111/j.1558-5646.2011.01427.x

[evl347-bib-0062] Nijhout, H. F. , D. A. Roff , and G. Davidowitz . 2010 Conflicting processes in the evolution of body size and development time. Philosophical Transactions of the Royal Society of London B: Biological Sciences 365:567–575. 20083633 10.1098/rstb.2009.0249 PMC2817141

[evl347-bib-0042] Paliwal, S. , A. C. Fiumera , and H. L. Fiumera . 2014 Mitochondrial‐nuclear epistasis contributes to phenotypic variation and coadaptation in natural isolates of *Saccharomyces cerevisiae* . Genetics 198:1251–1265. 25164882 10.1534/genetics.114.168575 PMC4224164

[evl347-bib-0043] Paranjpe, D. A. , D. Anitha , M. Chandrashekaran , A. Joshi , and V. K. Sharma . 2005 Possible role of eclosion rhythm in mediating the effects of light‐dark environments on pre‐adult development in *Drosophila melanogaster* . BMC Dev. Biol. 5:5. 15725348 10.1186/1471-213X-5-5 PMC554107

[evl347-bib-0044] Parry, G. D. 1983 The influence of the cost of growth on ectotherm metabolism. J. Theoret. Biol. 101:453–477. 6887950 10.1016/0022-5193(83)90150-9

[evl347-bib-0045] Pichaud, N. , J. W. O. Ballard , R. M. Tanguay , and P. U. Blier . 2013 Mitochondrial haplotype divergences affect specific temperature sensitivity of mitochondrial respiration. J. Bioenerg. Biomembr. 45:25–35. 23054075 10.1007/s10863-012-9473-9

[evl347-bib-0046] Raj, A. , S. A. Rifkin , E. Andersen , and A. van Oudenaarden . 2010 Variability in gene expression underlies incomplete penetrance. Nature 463:913–U984. 20164922 10.1038/nature08781 PMC2836165

[evl347-bib-0047] R Development Core Team . 2013 R: A language and environment for statistical computing. R Foundation for Statistical Computing, Vienna, Austria.

[evl347-bib-0048] Remold, S. K. , and R. E. Lenski . 2004 Pervasive joint influence of epistasis and plasticity on mutational effects in *Escherichia coli* . Nat. Genet. 36:423–426. 15072075 10.1038/ng1324

[evl347-bib-0049] Roff, D. A . 2002 Life history evolution. Sinauer, Sunderland, Massachusetts.

[evl347-bib-0050] Roff, D. A. , and D. J. Fairbairn . 2007 The evolution of trade‐offs: where are we? J. Evol. Biol. 20:433–447. 17305809 10.1111/j.1420-9101.2006.01255.x

[evl347-bib-0051] Schulte, P. M . 2015 The effects of temperature on aerobic metabolism: towards a mechanistic understanding of the responses of ectotherms to a changing environment. J. Exp. Biol. 218:1856–1866. 26085663 10.1242/jeb.118851

[evl347-bib-0052] Tennessen, J. M. , K. D. Baker , G. Lam , J. Evans , and C. S. Thummel . 2011 The *Drosophila* estrogen‐related receptor directs a metabolic switch that supports developmental growth. Cell Metab. 13:139–148. 21284981 10.1016/j.cmet.2011.01.005 PMC3072597

[evl347-bib-0053] Van Dyken, J. D. , and M. J. Wade . 2010 The genetic signature of conditional expression. Genetics 184:557–570. 19966065 10.1534/genetics.109.110163 PMC2828732

[evl347-bib-0054] Van Noordwijk, A. J. , and G. Dejong . 1986 Acquisition and allocation of resources ‐ their influence on variation in life‐history tactics. Am. Nat. 128:137–142.

[evl347-bib-0055] Wang, A. D. , N. P. Sharp , and A. F. Agrawal . 2013 Sensitivity of the distribution of mutational fitness effects to environment, genetic background, and adaptedness: a case study with *Drosophila* . Evolution 68:840–853. 24206451 10.1111/evo.12309

[evl347-bib-0056] Warton, D. I. , I. J. Wright , D. S. Falster , and M. Westoby . 2006 Bivariate line‐fitting methods for allometry. Biol. Rev. 81:259–291. 16573844 10.1017/S1464793106007007

[evl347-bib-0063] Yadav, P. , M. Thandapani , and V. K. Sharma . 2014 Interaction of light regimes and circadian clocks modulate timing of pre-adult developmental events in Drosophila. BMC Developmental Biology 14:19. 24885932 10.1186/1471-213X-14-19 PMC4040135

[evl347-bib-0057] Yang, H. , and Y. M. Yamashita . 2015 The regulated elimination of transit‐amplifying cells preserves tissue homeostasis during protein starvation in *Drosophila* testis. Development 142:1756–1766. 25968311 10.1242/dev.122663 PMC4440929

[evl347-bib-0058] Zhang, C. , K. L. Montooth , and B. R. Calvi . 2017 Incompatibility between mitochondrial and nuclear genomes during oogenesis results in ovarian failure and embryonic lethality. Development 144:2490–2503. 28576772 10.1242/dev.151951 PMC5536873

[evl347-bib-0059] Zhu, C‐T. , P. Ingelmo , and D. M. Rand . 2014 G×G×E for lifespan in *Drosophila*: mitochondrial, nuclear, and dietary interactions that modify longevity. PLoS Genet. 10:e1004354. 24832080 10.1371/journal.pgen.1004354 PMC4022469

