# Supplementary material for: Energy demand and the context‐dependent effects of genetic interactions underlying metabolism
Source: Evol Lett. 2018 Apr 3;2(2):102–13. doi: 10.1002/evl3.47 (PMC6121862; doi:10.1002/evl3.47)
Supplement: Supplementary file 1 — Table S1. Temperature‐dependent effects of mito‐nuclear interactions on development time are modified by the developmental photoperiod. Table S2. Developmental temperature and photoperiod can both independently modulate mito‐nuclear genetic effects on development time. Table S3. Gene‐environment interactions affect adult body mass. Table S4. Mito‐nuclear genetic effects on adult body mass are specific to females developed at 16°C. Table S5. Mito‐nuclear genetic effects on adult metabolic rate are specific to females developed at 16°C. Table S6. Mito‐nuclear interactions do not affect adult mass‐corrected metabolic rate. Table S7. Mito‐nuclear interactions do not affect metabolic plasticity (i.e., the Q 10 for metabolic rate) in adult females or males. Table S8. Mito‐nuclear interactions affect female, but not male, reproductive fitness. Figure S1. Weak effects of mito‐nuclear genotype on adult metabolic rate depend upon sex and measurement temperature. Figure S2. Adult male metabolic plasticity is not affected by mito‐nuclear genetic effects. [file EVL3-2-102-s001.docx]

**SUPPLEMENT**

**Energy demand and the context-dependent effects of genetic interactions underlying metabolism**

Luke A. Hoekstra, Cole R. Julick, Katelyn M. Mika, and Kristi L. Montooth

**Supplemental Tables S1-S8**

**Supplemental Figures S1-S2**

## Supplemental Table S1. Temperature-dependent effects of mito-nuclear interactions on development time are modified by the developmental photoperiod.

| Phenotype | Factor^1^ | numDF | denDf | *F*-value | *P*-value |
| --- | --- | --- | --- | --- | --- |
| 16°C Development time  (*N*=5261, 167 vials) | Photoperiod | 1 | 159 | 94.04 | <0.0001 |
|  | mtDNA | 1 | 159 | 47.29 | <0.0001 |
|  | Nuclear | 1 | 159 | 16.49 | 0.0001 |
|  | Photoperiod x mtDNA | 1 | 159 | 0.10 | 0.7535 |
|  | Photoperiod x Nuclear | 1 | 159 | 2.59 | 0.1092 |
|  | mtDNA x Nuclear | 1 | 159 | 19.46 | <0.0001 |
|  | Photoperiod x mtDNA x Nuclear | 1 | 159 | 25.43 | <0.0001 |
| 22°C Development time | Photoperiod | 1 | 153 | 688.29 | <0.0001 |
| (*N*=6191, 161 vials) | mtDNA | 1 | 153 | 1302.74 | <0.0001 |
|  | Nuclear | 1 | 153 | 885.38 | <0.0001 |
|  | Photoperiod x mtDNA | 1 | 153 | 332.88 | <0.0001 |
|  | Photoperiod x Nuclear | 1 | 153 | 193.61 | <0.0001 |
|  | mtDNA x Nuclear | 1 | 153 | 797.40 | <0.0001 |
|  | Photoperiod x mtDNA x Nuclear | 1 | 153 | 299.07 | <0.0001 |

^1^ Mixed-model ANOVA fit using restricted maximum likelihood and including culture vial as a random factor. Accompanies data presented in Figure 1. There was no evidence for a 4-way interaction between T_DEV_, photoperiod, mtDNA and nuclear genome (*F_1,312_* = 0.41, *P* =0.5244)

## Supplemental Table S2. Developmental temperature and photoperiod can both independently modulate mito-nuclear genetic effects on development time.

| Phenotype | Factor^1^ | numDF | denDf | *F*-value | *P*-value |
| --- | --- | --- | --- | --- | --- |
| 16°C, 12:12h Development time | mtDNA | 1 | 80 | 51.57 | <0.0001 |
| (*N*=2300, 84 vials) | Nuclear | 1 | 80 | 5.91 | 0.0173 |
|  | mtDNA x Nuclear | 1 | 80 | 0.38 | 0.5413 |
|  |  |  |  |  |  |
| 16°C, 24:0h Development time | mtDNA | 1 | 79 | 14.20 | 0.0003 |
| (*N*=2961, 83 vials) | Nuclear | 1 | 79 | 10.83 | 0.0015 |
|  | mtDNA x Nuclear | 1 | 79 | 30.13 | <0.0001 |
|  |  |  |  |  |  |
| 22°C, 12:12h Development time | mtDNA | 1 | 76 | 263.6 | <0.0001 |
| (*N*=3105, 80 vials) | Nuclear | 1 | 76 | 213.2 | <0.0001 |
|  | mtDNA x Nuclear | 1 | 76 | 104.0 | <0.0001 |
|  |  |  |  |  |  |
| 22°C, 24:0h Development time | mtDNA | 1 | 77 | 1059.82 | <0.0001 |
| (*N*=3086, 81 vials) | Nuclear | 1 | 77 | 663.62 | <0.0001 |
|  | mtDNA x Nuclear | 1 | 77 | 724.88 | <0.0001 |

^1^ Mixed-model ANOVA fit using restricted maximum likelihood and including culture vial as a random factor. Accompanies data presented in Figure 1.

**Supplemental Table S3.** Gene-environment interactions affect adult body mass.

| Phenotype | Factor | numDF | denDf | *F*-value | *P*-value |
| --- | --- | --- | --- | --- | --- |
| Adult mass^1^ | T_DEV_ | 1 | 317 | 486.085 | < 0.0001 |
| (*N*=333) | Sex | 1 | 317 | 807.744 | < 0.0001 |
|  | mtDNA | 1 | 317 | 1.549 | 0.2142 |
|  | Nuclear | 1 | 317 | 10.184 | 0.0016 |
|  | T_DEV_ x sex | 1 | 317 | 12.141 | 0.0006 |
|  | T_DEV_ x mtDNA | 1 | 317 | 1.351 | 0.2460 |
|  | Sex x mtDNA | 1 | 317 | 0.028 | 0.8674 |
|  | T_DEV_ x nuclear | 1 | 317 | 216.574 | <0.0001 |
|  | Sex x nuclear | 1 | 317 | 0.016 | 0.9001 |
|  | mtDNA x Nuclear | 1 | 317 | 6.078 | 0.0142 |
|  | T_DEV_ x sex x mtDNA | 1 | 317 | 0.152 | 0.6968 |
|  | T_DEV_ x sex x nuclear | 1 | 317 | 0.546 | 0.4604 |
|  | T_DEV_ x mtDNA x nuclear | 1 | 317 | 3.444 | 0.0644 |
|  | Sex x mtDNA x nuclear | 1 | 317 | 0.001 | 0.9734 |
|  | T_DEV_ x sex x mtDNA x nuclear | 1 | 317 | 0.016 | 0.1979 |

^1^ The dependent variable is the *ln*(*mass*) of groups of 10 adult flies developed at either 16°C or 22°C.

**Supplemental Table S4.** Mito-nuclear genetic effects on adult body mass are specific to females developed at 16°C.

| Phenotype | Factor | numDF | denDf | | *F*-value | *P*-value |
| --- | --- | --- | --- | --- | --- | --- |
| 16°C ♂ mass^1^ | mtDNA | 1 | | 82 | 0.059 | 0.809 |
| (*N*=86) | Nuclear | 1 | | 82 | 30.29 | <0.0001 |
|  | mtDNA x Nuclear | 1 | | 82 | 2.692 | 0.105 |
| 16°C ♀ mass^1^ | mtDNA | 1 | | 80 | 0.001 | 0.9777 |
| (*N*=84) | Nuclear | 1 | | 80 | 37.06 | <0.0001 |
|  | mtDNA x Nuclear | 1 | | 80 | 8.033 | 0.0058 |
| 25°C ♂ mass^1^ | mtDNA | 1 | | 77 | 1.090 | 0.300 |
| (*N*= 81) | Nuclear | 1 | | 77 | 96.79 | <0.0001 |
|  | mtDNA x Nuclear | 1 | | 77 | 1.187 | 0.279 |
| 25°C ♀ mass^1^ | mtDNA | 1 | | 78 | 1.931 | 0.169 |
| (*N*= 82) | Nuclear | 1 | | 78 | 66.00 | <0.0001 |
|  | mtDNA x Nuclear | 1 | | 78 | 0.101 | 0.752 |

^1^ The dependent variable is the *ln*(*mass*) of groups of 10 adult flies developed at either 16°C or 25°C.

## Supplemental Table S5. Mito-nuclear genetic effects on adult metabolic rate are specific to females developed at 16°C.

| Phenotype^1^ | | Genotype | | Common slope (95%CI) ^2^ | | Common slope  y-axis intercept (CI)^3^ | Shift along x- axis (mass) |
| --- | --- | --- | --- | --- | --- | --- | --- |
| 16°C ♂ Metabolic Rate | |  | | 0.885 (0.710,1.104) |  |  |  |
|  | | (*ore*);*OreR* | |  | 0.3281^a^ (0.1518, 0.5045) | no |  |
|  | | (*simw^501^*);*OreR* | |  | 0.3269^a^ (0.1482, 0.5056) | no |  |
|  | | (*ore*);*Aut* | |  | 0.3201^a^ (0.1410, 0.4991) | no |  |
|  | | (*simw^501^*);*Aut* | |  | 0.3283^a^ (0.1568, 0.5000) | no |  |
| 16°C ♀ Metabolic Rate | |  | | 1.098 (0.896,1.345) |  |  |  |
|  | | (*ore*);*OreR* | |  | 0.1569^a^ (-0.0733, 0.3872) | no |  |
|  | | (*simw^501^*);*OreR* | |  | **0.1328^b^** (-0.0957, 0.3613) | no |  |
|  | | (*ore*);*Aut* | |  | 0.1764^a^ (-0.0553, 0.4082) | no |  |
|  | | (*simw^501^*);*Aut* | |  | 0.1908^a^ (-0.0351, 0.4166) | no |  |
| 25°C ♂ Metabolic Rate | |  | | 1.134 (0.876,1.469) |  |  |  |
|  | | (*ore*);*OreR* | |  | 0.4480^a^ (0.2110, 0.6850) | no |  |
|  | | (*simw^501^*);*OreR* | |  | 0.4687^a^ (0.2308, 0.7065) | no |  |
|  | | (*ore*);*Aut* | |  | 0.4516^a^ (0.2107, 0.6925) | no |  |
|  | | (*simw^501^*);*Aut* | |  | 0.4650^a^ (0.2249, 0.7051) | no |  |
| 25°C ♀ Metabolic Rate | |  | | 1.077 (0.884,1.316) |  |  |  |
|  | | (*ore*);*OreR* | |  | **0.4622^a^** (0.2620, 0.6625) | no |  |
|  | | (*simw^501^*);*OreR* | |  | **0.4492**^a^ (0.2462, 0.6522) | no |  |
|  | | (*ore*);*Aut* | |  | 0.5355^b^ (0.3332, 0.7378) | no |  |
|  | | (*simw^501^*);*Aut* | |  | 0.5314^b^ (0.3289, 0.7339) | no |  |

^1^ Routine metabolic rate of adults of each sex measured at either 16°C or 25°C (data from the two development temperatures are pooled within measurement temperature). Accompanies data presented in Figure 2A and Supplemental Figure S1.

^2^ Common slope from a Type II model regression analysis of ln(metabolic rate) on ln(adult mass).

^3^ Different letters within a common slope denote significant differences in the y-intercept (i.e. the mass-specific metabolic rate) (*P* < 0.05).

**Supplemental Table S6.** Mito-nuclear interactions do not affect adult mass-corrected metabolic rate.

| Phenotype | Factor | numDF | denDf | | *F*-value | *P*-value |
| --- | --- | --- | --- | --- | --- | --- |
| 16°C ♀ MCMR^1^ | T_DEV_ | 1 | | 73 | 1.789 | 0.1852 |
| (*N*=81) | mtDNA | 1 | | 73 | 0.115 | 0.7356 |
|  | Nuclear | 1 | | 73 | 6.929 | 0.0103 |
|  | T_DEV_ x mtDNA | 1 | | 73 | 1.080 | 0.3022 |
|  | T_DEV_ x Nuclear | 1 | | 73 | 0.714 | 0.4010 |
|  | mtDNA x Nuclear | 1 | | 73 | 1.186 | 0.2796 |
|  | T_DEV_ x mtDNA x Nuclear | 1 | | 73 | 0.541 | 0.4646 |
|  |  |  | |  |  |  |
| 16°C ♂ MCMR^1^ | T_DEV_ | 1 | | 74 | 0.176 | 0.6759 |
| (*N*=82) | mtDNA | 1 | | 74 | 0.814 | 0.3698 |
|  | Nuclear | 1 | | 74 | 0.083 | 0.7735 |
|  | T_DEV_ x mtDNA | 1 | | 74 | 0.004 | 0.9476 |
|  | T_DEV_ x Nuclear | 1 | | 74 | 0.244 | 0.6226 |
|  | mtDNA x Nuclear | 1 | | 74 | 0.576 | 0.4502 |
|  | T_DEV_ x mtDNA x Nuclear | 1 | | 74 | 1.251 | 0.2670 |
|  |  |  | |  |  |  |
| 25°C ♀ MCMR^1^ | T_DEV_ | 1 | | 79 | 0.678 | 0.4127 |
| (*N*= 87) | mtDNA | 1 | | 79 | 0.060 | 0.8064 |
|  | Nuclear | 1 | | 79 | 34.678 | <0.0001 |
|  | T_DEV_ x mtDNA | 1 | | 79 | 1.233 | 0.2703 |
|  | T_DEV_ x Nuclear | 1 | | 79 | 0.358 | 0.5516 |
|  | mtDNA x Nuclear | 1 | | 79 | 0.001 | 0.9787 |
|  | T_DEV_ x mtDNA x Nuclear | 1 | | 79 | 0.023 | 0.8810 |
|  |  |  | |  |  |  |
| 25°C ♂ MCMR^1^ | T_DEV_ | 1 | | 78 | 5.949 | 0.0170 |
| (*N*= 86) | mtDNA | 1 | | 78 | 1.754 | 0.1892 |
|  | Nuclear | 1 | | 78 | 0.187 | 0.6666 |
|  | T_DEV_ x mtDNA | 1 | | 78 | 0.0003 | 0.9860 |
|  | T_DEV_ x Nuclear | 1 | | 78 | 6.676 | 0.0589 |
|  | mtDNA x Nuclear | 1 | | 78 | 0.061 | 0.8060 |
|  | T_DEV_ x mtDNA x Nuclear | 1 | | 78 | 0.082 | 0.7751 |

^1^ The dependent variable is the mass-corrected metabolic rate at each measurement temperature of groups of 10 adult flies developed at either 16°C or 22°C. See main text for calculation of mass-corrected metabolic rates. Accompanies data in Figure 2 and Supplemental Figure S2.

**Supplemental Table S7.** Mito-nuclear interactions do not affect metabolic plasticity (i.e., the *Q_10_* for metabolic rate) in adult females or males.

| Phenotype | Factor | numDF | denDf | *F*-value | *P*-value |
| --- | --- | --- | --- | --- | --- |
| ♀ MCMR^1^ | T_MEASURE_ | 1 | 152 | 945.888 | <.0001 |
| (*N*=168) | T_DEV_ | 1 | 152 | 0.035 | 0.8514 |
|  | mtDNA | 1 | 152 | 0.100 | 0.7519 |
|  | Nuclear | 1 | 152 | 41.32 | <.0001 |
|  | T_MEASURE_ x T_DEV_ | 1 | 152 | 1.947 | 0.1649 |
|  | T_MEASURE_ x mtDNA | 1 | 152 | 0.001 | 0.9817 |
|  | T_DEV_ x mtDNA | 1 | 152 | 0.397 | 0.5295 |
|  | T_MEASURE_ x nuclear | 1 | 152 | 16.015 | 0.0001 |
|  | T_DEV_ x nuclear | 1 | 152 | 0.044 | 0.8347 |
|  | mtDNA x Nuclear | 1 | 152 | 0.179 | 0.6731 |
|  | T_MEASURE_ x T_DEV_ x mtDNA | 1 | 152 | 2.030 | 0.1562 |
|  | T_MEASURE_ x T_DEV_ x nuclear | 1 | 152 | 0.823 | 0.3659 |
|  | T_MEASURE_ x mtDNA x nuclear | 1 | 152 | 0.202 | 0.6537 |
|  | T_DEV_ x mtDNA x nuclear | 1 | 152 | 0.029 | 0.8651 |
|  | T_MEASURE_ x T_DEV_ x mtDNA x nuclear | 1 | 152 | 0.203 | 0.6532 |
|  |  |  |  |  |  |
| ♂ MCMR^1^ | T_MEASURE_ | 1 | 152 | 751.276 | <.0001 |
| (*N*=168) | T_DEV_ | 1 | 152 | 5.861 | 0.0167 |
|  | mtDNA | 1 | 152 | 0.769 | 0.3818 |
|  | Nuclear | 1 | 152 | 0.231 | 0.6313 |
|  | T_MEASURE_ x T_DEV_ | 1 | 152 | 4.322 | 0.0393 |
|  | T_MEASURE_ x mtDNA | 1 | 152 | 2.348 | 0.1275 |
|  | T_DEV_ x mtDNA | 1 | 152 | 0 | 0.997 |
|  | T_MEASURE_ x nuclear | 1 | 152 | 0.080 | 0.7772 |
|  | T_DEV_ x nuclear | 1 | 152 | 3.860 | 0.0513 |
|  | mtDNA x Nuclear | 1 | 152 | 0.236 | 0.6278 |
|  | T_MEASURE_ x T_DEV_ x mtDNA | 1 | 152 | 0.003 | 0.9558 |
|  | T_MEASURE_ x T_DEV_ x nuclear | 1 | 152 | 2.430 | 0.1211 |
|  | T_MEASURE_ x mtDNA x nuclear | 1 | 152 | 0.004 | 0.9501 |
|  | T_DEV_ x mtDNA x nuclear | 1 | 152 | 0.020 | 0.8875 |
|  | T_MEASURE_ x T_DEV_ x mtDNA x nuclear | 1 | 152 | 0.463 | 0.4973 |

^1^ The dependent variable is the mass-corrected routine metabolic rate (MCMR) at each measurement temperature of groups of 10 adult flies developed at either 16°C or 22°C. See main text for calculation of MCMR. Accompanies data in Figure 2 and Supplemental Figure S2.

**Supplemental Table S8.** Mito-nuclear interactions affect female, but not male, reproductive fitness.

| Phenotype | Factor | numDF | denDf | | *F*-value | *P*-value |
| --- | --- | --- | --- | --- | --- | --- |
| Male fertility^1^ | mtDNA | 1 | | 107 | 4.236 | 0.042 |
| (*N*=115) | Nuclear | 1 | | 107 | 15.21 | 0.0002 |
|  | Block | 1 | | 107 | 1.541 | 0.217 |
|  | mtDNA x nuclear | 1 | | 107 | 0.185 | 0.668 |
|  | mtDNA x block | 1 | | 107 | 0.889 | 0.348 |
|  | Nuclear x block | 1 | | 107 | 4.128 | 0.045 |
|  | mtDNA x nuclear x block | 1 | | 107 | 1.435 | 0.234 |
|  |  |  | |  |  |  |
| Female fecundity^2^ | mtDNA | 1 | | 31 | 13.57 | 0.0009 |
| (*N*= 35) | Nuclear | 1 | | 31 | 120.8 | <0.0001 |
|  | mtDNA x nuclear | 1 | | 31 | 16.98 | 0.0003 |

^1^ Male fertility was scored as the number of offspring sired by individual males of each genotype per female mated.

^2^ Female fecundity was scored as the total number of eggs produced by individual females of each genotype over the course of 10 days. Data are from Meiklejohn *et al.* (2013).

**Supplemental Figure S1.** Weak effects of mito-nuclear genotype on adult metabolic rate depend upon sex and measurement temperature. Plots show routine metabolic rate as a function of mass on a log-log scale for pools of 10 male (A,C) or female (B,D) flies measured at 16°C (A,B) and at 25°C (C,D). Panel B reveals that the only evidence that mito-nuclear genotype affects adult metabolic rate is a modest, but significant, decrease in metabolic rate across masses specifically in females of the incompatible (*simw^501^*);*OreR* genotype when measured at 16°C (*P* < 0.05, Supplemental Table S5). For all other sex-T_MEASURE_ combinations, the (*simw^501^*);*OreR* genotype has a metabolic rate statistically similar to the (*ore*);*OreR* nuclear control genotype.

**Supplemental Figure S2.** Adult male metabolic plasticity is not affected by mito-nuclear genetic effects. A,B. Developmental reaction norms show robust thermal acclimation of male mass-corrected metabolic rate (MCMR) within measurement temperatures, although there were weakly significant effects of development temperature on male metabolic rate when measured at 25°C that depended weakly on nuclear genotype (T_DEV_, *F*_1,78_ = 5.949*, P* = 0.0170, T_DEV_ x Nuclear, *F*_1,78_ = 6.676*, P* = 0.0589; Supplemental Table S7). C, D. Thermal reaction norms show that the *Q_10_* for male MCMR is similar under both developmental temperatures. The mtDNA x nuclear interaction did not affect male MCMR at either measurement temperature (*P* > 0.45; Supplemental Table S6). Error bars are +/- 1 SEM.
